# Solventless Microextration Techniques for Pharmaceutical Analysis: The Greener Solution

**DOI:** 10.3389/fchem.2021.785830

**Published:** 2022-01-13

**Authors:** Heba M. Mohamed

**Affiliations:** Higher Colleges of Technology-Dubai Women’s Campus-Health Sciences, Dhabi, United Arab Emirates

**Keywords:** solventless extraction, microextraction, SPME, green analysis, pharmaceuticals

## Abstract

Extensive efforts have been made in the last decades to simplify the holistic sample preparation process. The idea of maximizing the extraction efficiency along with the reduction of extraction time, minimization/elimination of hazardous solvents, and miniaturization of the extraction device, eliminating sample pre- and posttreatment steps and reducing the sample volume requirement is always the goal for an analyst as it ensures the method’s congruency with the green analytical chemistry (GAC) principles and steps toward sustainability. In this context, the microextraction techniques such as solid-phase microextraction (SPME), stir bar sorptive extraction (SBSE), microextraction by packed sorbent (MEPS), fabric phase sorptive extraction (FPSE), in-tube extraction dynamic headspace (ITEX-DHS), and PAL SPME Arrow are being very active areas of research. To help transition into wider applications, the new solventless microextraction techniques have to be commercialized, automated, and validated, and their operating principles to be anchored to theory. In this work, the benefits and drawbacks of the advanced microextraction techniques will be discussed and compared, together with their applicability to the analysis of pharmaceuticals in different matrices.

## Introduction

As green analysis is becoming more significant, reduction of solvent use and method miniaturization gain most relevance in pharmaceutical analysis (Mohamed, 2015). Sample preparation is considered the cornerstone step for the green analytical procedure. There are different ways for more environment-friendly sample preparation, which mainly includes excluding or at least minimizing the amounts of reagents and solvents in the analysis. Besides, a green solvent is used, rather than petrol-based ones. Integration of automation is a very valuable principle in greening your procedure, which can lead to miniaturization of the used method (Płotka-Wasylka et al., 2013). As a result, novel solid-phase microextraction techniques (SPME, SBSE, MEPS, FPSE, ITEX-DHS, and PAL SPME Arrow) have been lately explored (Spieteluna et al., 2013). All of these innovative solutions enhance and enable the direct injection of the analytes into the separation unit, and accordingly, they require fewer solvents, time, and labor work (Płotka-Wasylka et al., 2015).

The SPME technique has been widely used in various areas such as food analysis, environmental analysis, bioanalysis, drug monitoring, and toxicology (Moein et al., 2015). Pharmaceuticals and biological materials are complex and may contain acids, bases, salts, biomolecules, and other additives with comparable properties to the analytes of interest that areactive ingredients and metabolites acting as interfering compounds (Ebele et al., 2017). Generally, sampling and sample preparation steps make up more than 80% of the total time of analysis, and these are very crucial steps in determining the success of the analysis of the compounds of interest in complex matrices such as biological samples, for instance, plasma, urine, sputum, oral fluid, and whole blood (Abuzar et al., 2017) or water, soil, and food samples (Zambonin and Aresta, 2021). Thus, it is not amplification when saying that the choice of a proper sample preparation method significantly affects the reliability and accuracy of the analysis in addition to its greenness and sustainability profiles.

The aim of this review is to report and compare the advantages and drawbacks of the recent techniques and devices used for the extraction and procedures for pharmaceuticals analyses in complex matrices, with the main goal being the reduction of solvent consumption, analysis time, and sample manipulation, in accordance with green analytical chemistry (GAC) concepts.

### Solid-Phase Microextraction

**FIGURE 1:**
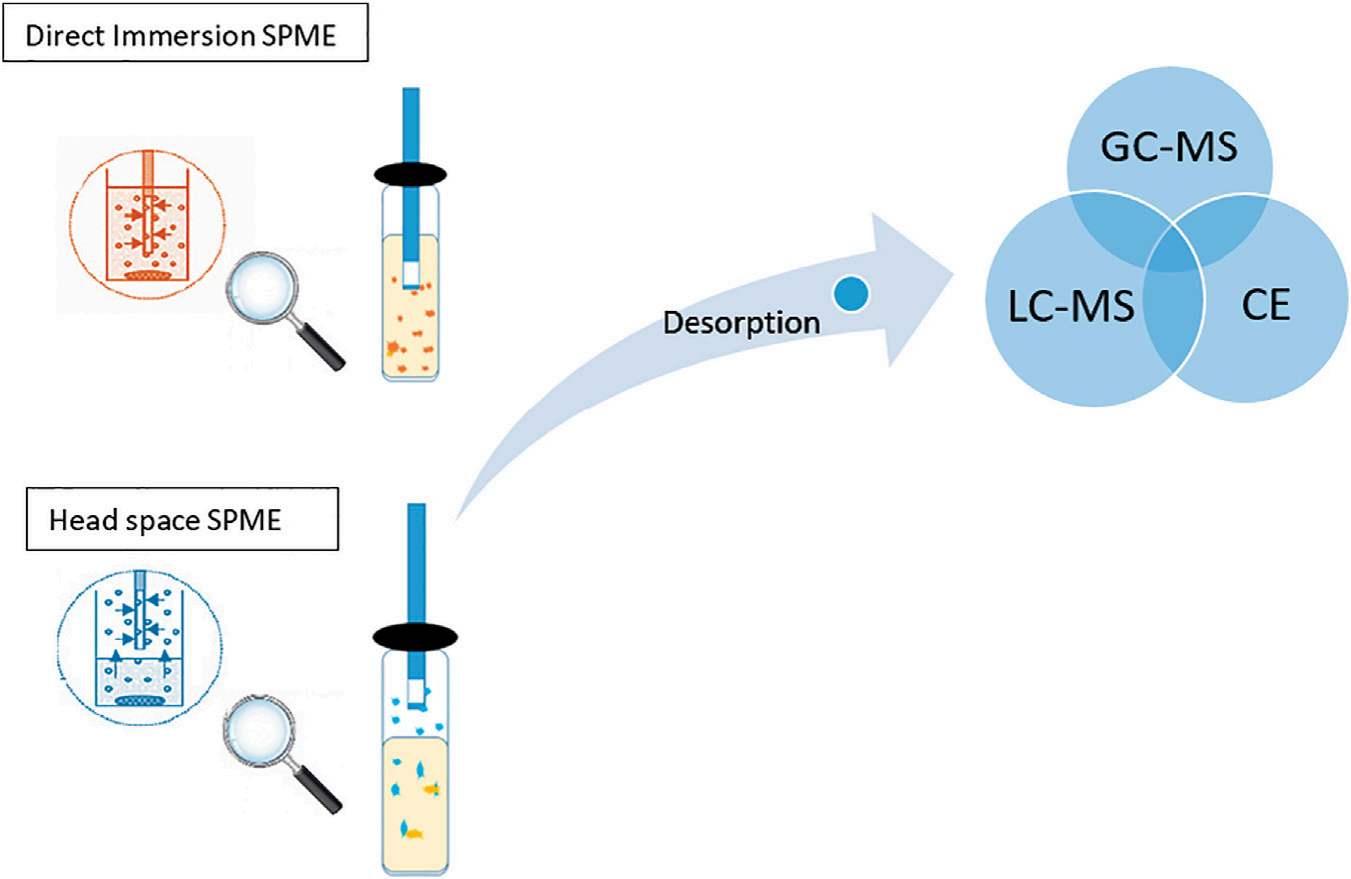
Process of extraction of analytes using direct immersion and headspace SPME that further coupled to chromatographic instrument (GC-MS or LC-MS or CE).

Solid-phase microextraction (SPME) presents a cornerstone for a new era of solventless extraction, miniaturization, and automation in pharmaceutical analysis. It assimilates sampling, extraction, and analyte pre-concentration into one single step, which results in low cost, reduction of labor, increased sensitivity, reduced carryover, and sample losses, and enhances the overall analytical process performance. Figure 1 shows the direct immersion (DI) and headspace (HS) SPME extraction and their direct coupling to the chromatographic system (Luo et al., 2011).

Nevertheless, one of the major downsides of SPME is the instability of the fibers: breaking and stripping of coatings that can significantly reduce their overall lifetime (Reyes-Garcés et al., 2018). Using HS extraction, rather than the DI, has improved the lifetime, yet it is still considered a drawback (Płotka-Wasylka et al., 2015). Another negative point of SPME is the reproducibility that mainly results from various factors related to batch-to-batch variation of fiber coatings, low thermal stability of the fibers, short expiry date, and small selectivity. These factors trigger the improvement of materials used for SPME. In general, the DI-SPME has better reproducibility, but it does not allow changes to the sample matrix, for example, change in pH (Płotka-Wasylka et al., 2015).

#### Fiber Solid-Phase Microextraction and Capillary Solid-Phase Microextraction

The commercially available fibers can be classified into non-bonded, bonded, partially cross-linked, and highly cross-linked phases, which all differ in their degree of stability with organic, polar, and non-polar solvents. The physicochemical properties and coating thickness strongly affect the distribution of analytes between the sample matrix and the extraction phase, which in turn influences the efficiency of extraction, selectivity, and reproducibility of the analysis. Factors to be considered while selecting of the fibers are molecular weight, polarity, and volatility of target analytes (Spietelun et al., 2010). To enhance the selectivity and applicability of solid-phase microextraction (SPME), different coating techniques have been explored and utilized.

#### Conventional SPME Fiber Coatings

Polydimethylsiloxane (PDMS), polydimethylsiloxane/divinylbenzene (PDMS/DVB), divinylbenzene/carboxen/polydimethylsiloxane (DVB/CAR/PDMS), carbowax/divinylbenzene (CW/DVB), and carbowax/templated resin are used for conventional SPME fibers in both HS and DI modes (Portillo-Castillo et al., 2018). The main problem for the conventional coating is that they are mostly restricted to non-polar or relatively non-polar analytes with relatively low commercial availability (Souza et al., 2013).

#### Ionic Liquid–Based SPME Fiber Coatings

Ionic liquids are common alternative fiber coating materials. IL-SPME fibers are mostly manufactured by immersion–agglutination techniques for both fused silica and metallic supports. Sometimes, a combination of ILs and adhesive or binder is used to obtain thicker and more resistant coatings. One of the superior advantages is their high viscosity that helps to acquire enhanced coatings and improve film homogeneity and integrity. Moreover, due to the high thermal stability of ionic liquids, the fibers are more resistant with higher half-lives than the conventional ones. In addition, the hydrophobicity of the ionic liquids has to be considered while preparing the coating material. Better extraction efficiency is due to the easier analyte diffusion from bulk matrix samples to SPME fibers with the aid of the ionic liquid. The most commonly used ionic liquids for SPME coating are the ones containing imidazolium cations combined with different anions (Portillo-Castillo et al., 2018).

**FIGURE 2:**
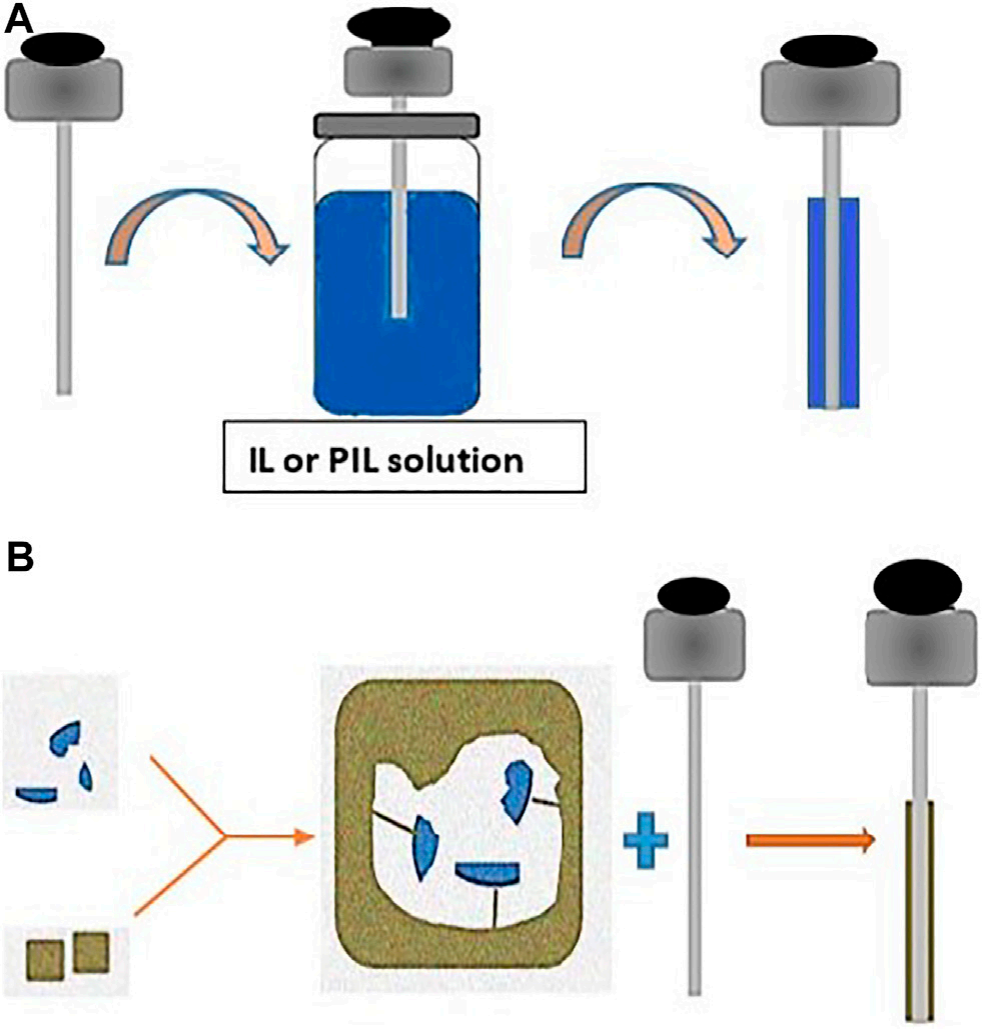
**(A)** Immersion–agglutination coating procedure to obtain IL- or PIL-coated SPME fibers. **(B)** MIP fabrication and coating of SPME fibers.

Polymeric ionic liquids (PILs) are polymeric analogs of ILs; they have the same selectivity and solvation power. However, their viscosity and thermal stability are much higher. As a result, PIL-based coatings have been used for SPME to improve their thermal, mechanical, and chemical resistance. Additionally, it extended their applicability to various samples and different analytes. Afterward, the functionalized PIL-SPME was used to further expand their application. Figure 2A shows an immersion–agglutination coating procedure to obtain IL/PIL-SPME fibers.

In further modification, monolithic SPME fibers are prepared with different materials such as polymers or graphene in a form of very thin rods. Their key advantages are simple preparation, flexibility, and solvent resistance, and they show higher mass transference rates than traditional fibers. Monolithic fibers can be used for direct immersion–solid-phase microextraction (DI-SPME) extractions due to their physical and chemical characteristics (Mei et al., 2014).

Another alternative approach is the multiple monolithic fiber SPME (MMF-SPME); it is based on using several monolithic fibers bound together in a device. The available spaces between the fibers allow sample convection and improve the mass transfer (Mei et al., 2014). Recently, PILs have been used as MMF-SPME fiber coating.

#### Molecularly Imprinted Polymer SPME Fiber Coatings

Molecularly imprinted polymers are obtained by monomer polymerization with a cross-linking agent using a selected template molecule, mainly through free radical polymerization. The role of the template molecules is to organize the monomer functional groups and outline the properties of binding sites (Zhang et al., 2013). Among the common templates that are used for the molecularly imprinted polymers are drugs, amino acids, proteins, carbohydrates, nucleotides, and hormones. Cross-linking agents mainly function to control the morphology of the matrix and to stabilize the binding site; examples of the common cross-linking agents are ethylene glycol dimethacrylate, trimethylolpropane trimethacrylate, and others. Chloroform, dichloromethane, and acetonitrile are the commonly used solvent in the manufacturing process to maintain all components in only one phase (Vasapollo et al., 2011).

Figure 2B presents a scheme of the fabrication basic principle of MIP and an example of an SPME fiber coating process. MPIs are mainly used for coating SPME-fused silica or metallic fibers. MIP-SPME fibers can be used for direct immersion due to their high solvent resistance and wide pH stability.

#### Carbon-Based SPME Fiber Coatings

Carbon nanotubes (CNTs), graphene, graphene oxide (GO), and fullerenes are very widely used in sample extraction techniques. Their high superficial area ensures a high extraction capacity (Souza, Risticevic, and Pawliszyn 2013). Graphene (mostly GO) is commonly used in SPME fiber coating for its high surface area and mechanical, chemical, and thermal stability. Being easily functionalized, it offers greater flexibility in extracting different compounds with different natures. It can be prepared by immersion of a stainless steel wire in a graphene adhesive suspension that results in a 6- to 8-μm-thick coating film or by sol-gel procedures. Graphene-based fibers yield 1.5 times higher extraction efficiencies than poly(dimethylsiloxane (PDMS) and poly(dimethylsiloxane/divinylbenzene (PDMS/DVB) commercial fibers; this can be related to their porous surface and their strong affinities to analytes (Portillo-Castillo et al., 2018). Graphene-based SPME fiber was used for extraction of some UV filters that are frequently used in cosmetics, sunscreens, and personal care products (Zhang and Lee 2012a). Multi-walled nanotubes (MWNTs) functionalized with polyethylene glycol were used for coating a fused silica fiber via a sol-gel process. CNTs and MWNTs were used to extract ibuprofen, naproxen, and diclofenac from water samples; it was stable at high temperatures up to 320°C and used for at least 150 extraction cycles (Sarafraz et al., 2012).

#### Other Materials for SPME Fiber Coatings

Some inorganic materials can be used in SPME fibers due to their good thermal, mechanical, and chemical stability. They also show good selectivity due to their electronic structures, hydrophobic interactions, electrostatic attractions, or covalent bond formation. Ti–TiO_2_–ZrO_2_ fiber was used for PPCP analysis, 2-hydroxy-4-methoxybenzophenone, 2-ethylhexyl-4-methoxycinnamate, 2-ethylhexyl-4-(N,N-dimethylamino) benzoate, and ethylhexyl salicylate were extracted by DI of the Ti–TiO_2_–ZrO_2_ fiber into the samples with good performance (Li et al., 2020). Gold nanoparticles (C8-S-AuNPs/SS) have also been used for SPME fiber coating and applied to UV filters and phthalate ester (Yang et al., 2017). Metal oxide nanosheets, such as, TiO_2_ nanosheets and Zn–ZnO nanosheets were used. Conducting polymers (CPs), namely, polypyrrole, polyaniline, and its derivatives have been used as SPME fiber coating. CPs are rigid molecules producing fibers with adequate hardness and sturdiness and show food adsorption capacity (Bagheri, Ayazi, and Naderi 2013). Metal–organic frameworks (MOFs) are also used as a coating material; they are porous materials formed by metal ion clusters connected by organic ligands (Tian et al., 2013).

### In-Tube Solid-Phase Microextraction/In-Tube Extraction Dynamic Headspace ITEX-DHS

This technique uses an open tubular capillary as an SPME device and can be coupled with liquid chromatography (LC). It can also be coupled to GC using a GC capillary tube (Kataoka, 2005). In in-tube SPME, the extraction, desorption, and injection can be automated using a standard autosampler. The automation has advantages like shorter analysis time, more accurate, and precise results. The analyte extraction depends on the polarity of the capillary coating, the number and volume of draw/eject cycles, and pH (Kataoka, 2005). In addition, the internal diameter, length, and film thickness of the column have to be chosen carefully. For optimum extraction, the capillary column length should be around 50–60 cm; if the extraction is lower, efficiency is reduced, and above this level, peak broadening can be observed (Kataoka, 2005).

ITEX-DHS grants many advantages compared to the previously described techniques, especially having an external heater unit that allows desorption to be done independently of the injector temperature (Parodi et al., 2019). So far, most of the volatile organic compounds (VOCs) have been analyzed using ITEX-DHS, although many commercial sorbent materials are available. This could be related to the fact that ITEX-DHS is only suitable for HS extraction (Kataoka, 2005).

### Solid-Phase Dynamic Extraction

It is an inside-needle technique for vapor and liquid sampling (Ke˛dziora-Koch and Wasiak, 2018). It is composed of stainless steel needles (8 cm) coated with a 50-mm film of PDMS and 10% activated carbon on which the analytes are concentrated. The robustness of the capillary is the main advantage of SPDE over SPME. SPDE has been successfully applied to the analysis of volatile compounds, pesticides, and some drugs. Nevertheless, one of SPDE’s disadvantages is the carryover, since the analytes tend to remain in the inside needle wall followed by heat desorption in the GC injection port (Luo et al., 2010).

### Microextraction in a Packed Sorbent

MEPS is considered a potential extraction method that allows for little solvent consumption, small sample volume (10–250 µl) that can be directly injected without additional treatments into GC, LC, or MS and without any modification of the instrument. Another green key point is that it only requires a small amount of sorbent, so only relatively small amounts of solvents are needed for the elution process of the different analytes (Moein et al., 2015). The process for MEPS includes conditional step, sample loading, sample washing, sample elution, and a final MEPS cleaning step. The MEPS device has multiple uses and variable applications, such as, biologicals and environmental and food applications, yet in environmental applications, where the sample volume is high, MEPS shows some drawbacks (Silva et al., 2014).

**FIGURE 3:**
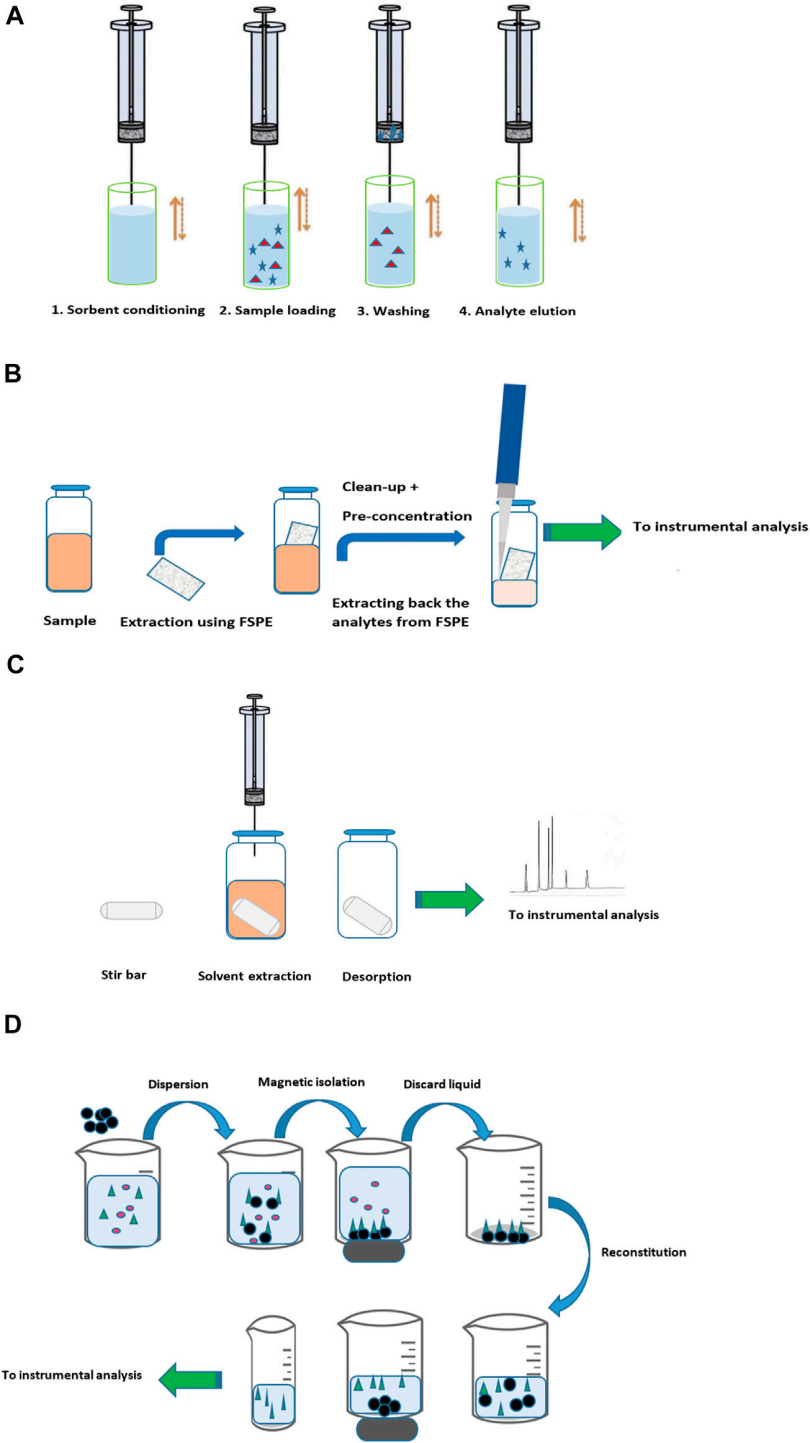
**(A)** General procedure applied in MEPS extraction. **(B)** General procedure applied in FPSE. **(C)** General procedure applied in SBSE. **(D)** General procedure applied in magnetic nanoparticle extraction.

Several types of packing materials are available; unmodified silica, C2, C8, C18, polystyrene–divinylbenzene (PS-DVB), porous graphitic carbon, MIPs, monoclonal antibodies (mAbs) for immunoaffinity sorbent production, and other commercial ones, as recently reviewed (Yang et al., 2017). In order to improve the reproducibility of the MEPS devices, they are coupled to syringes as they help in the reproducibility of the flow rates. Figure 3A shows the general procedure applied in MEPS extraction.

### Fabric Phase Sorptive Extraction Procedures

It is a relatively new method, developed by Kabir and Furton (2014), that overcomes the drawback of MEPS as it is effectively used for small- and large-volume samples and could be practically applied in all fields (environmental, biological, food, toxicological, pharmaceutical, and quality control). Recently, the inventors introduce adjustments for FPSE not to either require matrix modifications or clean up (Kabir et al., 2018). FPSE significantly simplifies the sample preparation workflow compared to other available techniques. Figure 3B shows the general procedure applied in FPSE.

### Stir Bar Sorptive Extraction

A stir bar with a polymer coat is used to extract the analytes typically by direct immersion; the analytes are extracted and pre-concentrated when the SBSE spins inside the solution. Afterward, the analytes are desorbed using a thermal desorption unit coupled to gas chromatography or by solvent desorption busing a small volume of a suitable organic solvent. It was developed to increase the extraction sensitivity by incorporating substantially higher sorbent loading. As it eliminates the use of solvents and reduces the exhaustive and time-consuming sample preparation step, SBSE simply satisfies the GAC requirements. Only two phases are available, PDMS and poly(ethylene glycol) in PDMS, to be coated onto a glass-coated magnetic bar, and due to their high viscosity, they slow down analyte diffusion during extraction that negatively impacted the extraction sensitivity in SBSE (Płotka-Wasylka et al., 2015). The availability of only two phases is considered as the major drawback of the method. The SBSE is considered as a direct and independent sample preparation device, it can simply operate without the need of an external magnet by diffusing the sample matrix on a magnetic stirrer where both extraction and pre-concentration are directly done. Figure 3C shows the general procedure applied in SBSE.

### Magnetic Nanoparticle Extraction

Recently, magnetic nanoparticles have been used for selective extraction of trace species from complex matrices. The dominant advantages of this innovative technique are it is possible to retain the adsorbed analytes directly in the tube, clean away the matrix and the interference compounds, and analyze the trace species in the extract directly. In addition, the use of strong magnet ensures no loss of analytes in the washing step and the possibility of having a great pre-concentration factor for trace analyses. This technique is reported to be used in food analysis (Hernández-Hernández et al., 2017), for drugs in biological matrices (Vasconcelos and Fernandes, 2017), or in other research fields (Ríos and Zougagh, 2017). Figure 3C shows the general procedure applied in magnetic nanoparticle extraction.

### PAL SPME Arrow

**FIGURE 4:**
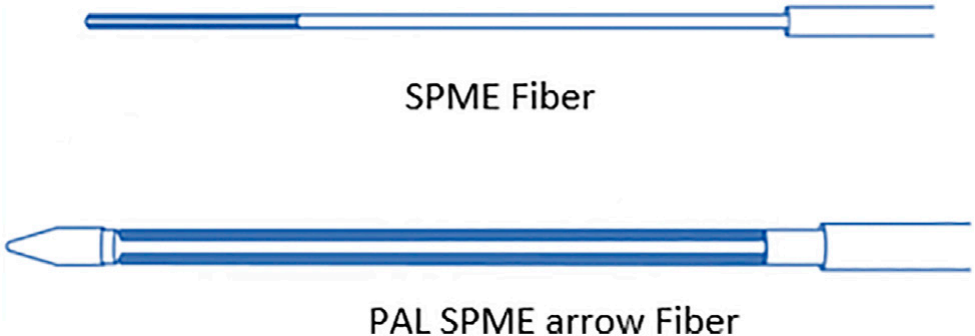
Difference between classical SPME fiber and the PAL SPME arrow.

PAL SPME Arrow was recently introduced to overcome the drawbacks of SPME and SBSE. It combines trace-level sensitivity with high mechanical robustness. Contrary to the classical SPME fibers, the Arrow design fully shields the sorptive material, reducing adverse effects and losing of analytes during transfer processes. Additionally, PAL SPME Arrow has a larger sorbent volume, providing a higher extraction capacity than SPME, as seen in Figure 4. In contrary to SBSE, it can be fully automated. It was used and compared to SPME in extracting polycyclic aromatic hydrocarbons (PAHs) from the freely dissolved fraction in laboratory water and groundwater using polydimethylsiloxane (PDMS) as common sorption phase material via direct immersion (Kremser et al., 2016). Table 1 compares the advantages and drawbacks of these microextraction techniques.

**TABLE 1:** Advantages and limitations of some green solid phase-based extraction techniques.

Microextraction technique	Advantages	Disadvantages
Solid phase microextraction (SPME)	No need for solvents	Robustness of fiber coatings
	Rapid, simple and sensitive	Stationary phase of limited range
	Used for polar and non-polar analytes	In-between batch variations
	Applicable with wide range of matrices	
	Compatible with different separation and detection systems	
	Suitable for headspace and immersion modes	
In-tube extraction dynamic headspace (ITEX-DHS)	High sorption capacity	Stationary phase of limited range
	Many available commercial sorbent materials	
	External heater unit allows independent desorption	
Solid-phase dynamic extraction (SPDE)	More sensitive than SPME	Carryover; analytes might remain on the needle inner wall
	Robust	
	Shorter extraction time than SPME	Stationary phase of limited range
	Smaller sample size than SPME	
		More complicated analytical process
Microextraction in a packed sorbent (MEPS)	Short procedure time	Clogging of the barrel insert and needle
	Applicable with wide range of matrices	Not very suitable for processing large volume samples
	More simple analytical procedure	Limited range of sorbents available
	Reuse sorbents many time	
	Economical	
Fabric phase sorptive extraction procedures (FPSE)	No special equipment or set-up is needed, flexible technique	Longer extraction time
	Stable even in harsh chemical environment (pH 1–13)	
	High primary contact surface area (efficient extraction)	
	Low solvent required for quantitative desorption	
	Low risk of cross-contamination	
Stir bar sorptive extraction (SBSE)	Lower detection limit than SPME	High matrix effects
	Compatible with different separation and detection systems	Limited number of commercially available coatings
	High thermal and chemical stability of stir-bar coatings	Requires high control of extraction conditions
	Suitable for headspace and immersion modes	High enrichment factor only for non-polar analytes
	Higher enrichment factor than SPME	Possibility of bleeding at even relatively during thermal desorption
Magnetic nanoparticle extraction	Good cleanup of matrix and the interference compounds	Particle agglomeration that leads to low extraction efficiency
	No loss of analytes	Oxidation of magnetic cores
PAL SPME Arrow	Higher extraction capacity than SPME	Limited mechanical stability
	High mechanical robustness	Small phase volumes of the fibers
	Trace-level sensitivity	
	Can be fully automated	

## Extraction Efficiencies of SPME Techniques

Despite the practical applicability of SPME in sampling procedures, modifications to improve the extraction efficiencies are still a research aim. In general, the extraction efficiencies for SPME will depend on extraction conditions, that is, temperature, extraction time, and mixing method, ionic strength of solutions, sample pH, stationary phase volume, headspace phase volume, the volume of the sample, and fiber material (Rutkowska et al., 2014).

It could be improved by adding salt to the sample for example sodium chloride and sodium sulfate (salting out), pH or temperature changes (as it affects the partitioning of the targeted analytes, due to the different pKa values); agitating the sample by stirring, for example, it can enhance the extraction efficiency in non-equilibrium situations (Fiorini et al., 2015).

Other solutions related to the use of unique phases have been developed to improve extraction efficiency, for instance, using inner coating with polypyrrole polymers for the commercially fused silica capillary (Portillo-Castillo et al., 2018). In addition, optimum column length is important for the efficiency of extraction. Other techniques include wire-in-tube or fiber-in-tube SPME, which improves extraction efficiency while extending the method to microscale applications.

In SBSE, the sample volume and the speed of stirring impact the extraction efficiency, and the usual stirring times for equilibration are between 30 and 60 min (Pang et al., 2017). The introductions of SPME–trap and SPME–trap with multi-step enrichment (MSE) are improvements in sample extraction and lower detection limits (Emmons et al., 2019).

Since the extraction efficiency can be affected by a high number of factors, the role of each factor and its interactions have to be studied for better extraction outcomes. For this purpose, formal design of experiments (DoE) can provide a fast, efficient, and cost-effective approach that is superior to that achievable by the one-factor-at-a-time (OFAT) methodology. Two broad groups of experimental designs are used: simple linear models (screen from two to 6–8 factors) and the more complicated quadratic models to describe the response as a function of two to four factors by applying the response surface methodology (RSM) (Marrubini et al., 2020). There are many pieces of software used for DoE computations, namely, Chemometric Agile Tool (CAT), Design Expert, Minitab^®^ Statistical Software, JMP, and MATLAB. Marrubini and coworkers reviewed articles published in the period from 2009 to 2019 that used microextraction techniques and applied DoE. They included some points that represent the least information needed to describe and report a DoE study: the experimental plan and experimental matrix, the model equation and model adequacy checking (R^2^, adjusted R^2^, residuals, analysis of variance (ANOVA), *t*-test of the coefficients, normal probability plot, inflation factors), the model validation and finally the response surfaces plots, interpretation, and discussion of the role of the factors (Marrubini et al., 2020).

## Selection Criteria for the Microextraction Technique

The selection of the optimal microextraction technique can be an overwhelming task for the researchers, and it highly depends on the specific analytical problem. The characteristics of the analytes are very crucial to determine the most suitable extraction technique, and the analytes’ partitioning constants can help predict the suitability of the DI or HS extraction mode (Parodi et al., 2019). Another factor to be considered is the volatility of the analytes, if the analytes are sufficiently volatile, HS is always preferred over DI extraction. Using HS with volatile analytes allows exclusion of more interferences, faster equilibrium due to the diffusion boundary layer being thinner, and prolonged lifetime of the sorbent material (Płotka-Wasylka et al., 2015). Both DI and HS sampling modes of SPME are used extensively in SPME–GC application. HS extraction can be performed with all the techniques previously described; however, it is best fit with the dynamic extraction techniques like ITEX-DHS. While DI–SPME is more suitable for gaseous or simple liquid sample matrices, HS–SPME is preferentially used for extraction from complex liquid and solid samples. For very dirty samples, the HS mode is the most suitable, yet if the analysis becomes more complicated, pH adjustment or salt addition should be performed to facilitate the transfer of the target compounds to the headspace. HS also is more suitable to analyze highly volatile and low-polar analytes in contrary to DI extraction. Commonly, for SPME–LC applications, DI is used as the analytes of interest are not sufficiently volatile. PAL SPME Arrow, MEPS, and SBSE are the recommended techniques, especially when coupled to GC. Better sensitivities are achieved by SBSE due to the higher phase volume available. Nevertheless, it still requires a significantly longer time to reach equilibrium in addition to substantial matrix effects (Płotka-Wasylka et al., 2015). The limiting spectrum of analytes for SBSE is mainly due to the limited availability of the coating material. If the optimal phase is not available in the SBSE design, PAL SPME Arrow is the recommended alternative. Table 1 summarizes the advantages and limitations of these green microextraction techniques.

## Applications

A wide application range for pharmaceutical extractions using solventless techniques is reported, which includes analysis of pharmaceuticals in environmental, water, food, and biological matrices. All applications would never be possible to summarize; yet in Table 2, some solventless extraction techniques used for pharmaceutical applications in different matrices are presented.

**TABLE 2:** Applications of the microextraction techniques discussed in this review for determination of pharmaceuticals in different matrices.

Application	Analyte	Extraction method	Matrix	LOD/LOQ (ng/ml)	Recovery	Analysis method	References
Environmental	Antioxidants, UV filters, and fragrances	SPME(PDMS)	Tap water	5–8,500	nr	GC-MS	Basaglia et al. (2011)
	Estriol, estradiol, ethynylestradiol, estrone, progesterone, medroxyprogesterone, levonorgestrel, and norethindrone	SBSE	Water (tap water and raw	0.5 × 10^–3^–1.0	556–96%	MS	Vo Duy et al. (2012)
			Wastewater				
	Triclosan, triclocarban, and their four related transformation products	SPME	Water	0.06–0.21	81.54–102.32%	HPLC-DAD	Shen et al. (2012)
				0.12–0.73			
	Triclosan and bisphenol-A	SPME (CW/TPR)	Tap, river and	1	71–111	HPLC-DAD	Kim et al. (2013)
	Chlorophenols and mefenamic acid		Wastewater	2–9			
	Gemfibrozil			3			
	Steroids	SPME (PDMS/DVB)	Local source water	30–2,000	84.9–103%	GC-MS/MS	Chopra et al. (2014)
	Mesterolone						
	Methandriol						
	Estrone						
	Estradiol						
	Androstenedione						
	Eticholan-3–17-one						
	DES						
	Prasterone						
	Sulfonamides, sulfacetamide, sulfadiazine, sulfathiazole, sulfamethazine, sulfamethoxypyridazine, and sulfamethoxazole	MEPS(C8)	Wastewater	5–200	88–109%	HPLC-DAD	Salami and Queiroz, (2014)
	Flufenamic acid and mefenamic acid	SPME	Water	6 × 10^−5^–1.24 × 10^−3^	85.1–110.8%	GC-MS	Huang et al. (2015)
	Flurbiprofen			2.2 × 10^−4^–4.13 × 10^−3^			
	Clofibrate						
	Ketoprofen						
	Naproxen						
	Tolfenamic acid						
	Gemfibrozil						
	Estriol, 17β-estradiol, testosterone, ethinylestradiol, estrone, progesterone, and mestranol	SPME-fiber	Water	nr	75.6–116%	HPLC	Liao et al. (2016)
	Androgens and progestogens	FSPE (sol-gel PTHF)	Water, urine	1.7 × 10^−3^–0.264 (water samples)	83.8–103.9% water	UHPLC–MS/MS	Guedes-Alonso et al. (2016)
	Norethisterone			8.9 × 10^−3^–0.132 (urine samples)	81.9–120% urine		
	Norgestrel						
	Megestrol acetate						
	Progesterone						
	Boldenone						
	Nandrolone						
	Testosterone						
	DHEA						
	Androsterone						
	Androstenedione						
	Penicillins (penicillin G, penicillin V, oxacillin, cloxacillin, dicloxacillin, and nafcillin)	SPME (Al-MOF)	River water	0.06–0.26	80.8–90.9% in water	LC–MS or LC–UV	Lirio et al. (2016)
					81.1–100.7% in milk		
	Abacavir	SPME-fiber	Surface waters and wastewater	0.010–0.013	88–99%	LC-MS	Terzopoulou et al. (2016)
				0.033			
	Chlorophenols and eleven estrogenic compounds	SPME	Surface water	8.8 × 10^−3^–42.9 × 10^−3^	69–108%	GC-MS	Yuan et al. (2017)
	β-Estradiol	SBSE	Water	nr	71.4–83.2%		Suazo et al. (2017)
	3-(4-metylbenzylidene)camphor						
	Carbamazepine						
	Ibuprofen						
	2,4-dihidroxybenzophenone						
	Triclosan	SPME-Fiber (PDMS)	Seawater	0.111, 0.177, 0.088	nr	LC-MS/MS	Santos et al. (2018)
	Bisphenol A						
	17α-Ethynylestradiol						
	4-Chloro-1-naphthol	SBSE	Wastewater, pool water	0.034	87.4–141.3%	HPLC	Jillani et al. (2020)
				0.400			
Biological (fluids and tissues)	NSAIDs	MEPS (C18)	Plasma, urine	30–100	nr	HPLC-PDA	Locatelli et al. (2014)
	Furprofen, indoprofen, ketoprofen, fenbufen, flurbiprofen, indomethacin, and ibuprofen						
	Selected estrogens	FSPE (sol-gel PTHF)	Urine	0.036	88.7–98.0%	HPLC-FLD	Kumar et al. (2014)
	α-17-ethynylestradiol			0.020			
	β-Estradiol			0.042			
	α-Bisphenol A						
	Ciprofloxacin and levofloxacin, two fluoroquinolones	MEPS (C18)	Sputum	17–50	>80%	HPLC-PDA	Locatelli et al. (2015)
	Cocaine, amphetamines, natural and synthetic opioids, and hallucinogens (AMP, MAMP, MDA, MDMA, and MDEA)	MEPS	Oral fluid	1, 1, 1, 0.5 & 0.5	>60%	LC-MS/MS	Montesano et al. (2015)
	Estriol, 17β-estradiol, testosterone, ethinylestradiol, estrone, progesterone, and mestranol	SPME-fiber	Urine	nr	75.6–116%	HPLC	Liao et al. (2016)
	Benzodiazepines	FSPE (sol-gel PEG)	Blood serum	30	nr	HPLC	Samanidou et al. (2016)
				10			
	Abacavir	SPME-fiber	Urine	43.9 × 10^−3^	88–99%	LC-MS	Terzopoulou et al. (2016)
	12 azole drugs (bifonazole, butoconazole, clotrimazole, econazole, itraconazole, ketoconazole, miconazole, posaconazole, ravuconazole, terconazole, tioconazole, and voriconazole)	MEPS (C18)	Plasma, urine	0.23 and 0.37	88.5–99.2%	HPLC-DAD	Campestre et al. (2017)
	Trans,trans-muconic acid	MIP-MEPS	Urine	50	89.8–91.6%	HPLC-UV	Soleimani et al. (2017)
				15			
	Statins	MEPS (C18)	Plasma	10–20	nr	UHPLC-MS/MS	Ortega et al. (2017)
	Drugs of abuse	MEPS C8/SCX	Plasma	5–10	80–104%	UPLC	Fernández et al. (2017)
	Morphine						
	Methylone						
	6-AM						
	Mephedrone						
	BEG						
	Cocaine						
	MDPV						
	Cocaethylene						
	EDDP					
	Methadone					
	Voriconazole	SPME-MS	Human plasma	3–6	nr	Coated blade spray-MS	Tascon et al. (2017)
	Ciprofloxacin	FSPE (sol-gel Carbowax^®^ 20 M)	Whole blood Plasma	250 (10)	nr	HPLC-PDA	Kabir, et al. (2018)
	Sulfasalazine		Urine	110 (30)			
	Cortisone			100 (30)			
	Cyclosporine	SPME-MS	Whole blood	3.0	nr	Coated blade spray-MS/MS	Gomez-Ríos et al. (2018)
	Tacrolimus		Whole blood	0.3			
	Sirolimus		Whole blood	1.0			
	Everolimus		Whole blood	0.3			
	losartan and valsartan	SBSE	Human plasma	7.0	98–117%	LC-MS	Babarahimi et al. (2018)
				27.0			
	Methylphenidate	SPME	Human heparin plasma	nr	nr	TD-ESI/MS	Wang et al. (2018)
	Doxorubicin	SPME	Lung tissue		103.2%	LC-MS/MS	Roszkowska et al. (2018)
	Antibiotics and their metabolites (amoxicillin, cefotaxime, ciprofloxacin, clindamycin, metronidazole, amoxycilloic acid, 4-hydroxyphenyl glycyl amoxicillin, desacetyl cefotaxime, 3-desacetyl cefotaxime lactone, ciprofloxacin N-oxide, N-demethyl clindamycin, clindamycin sulfoxide, and hydroxy metronidazole	SPME-C18 fiber	Human whole blood and tissue samples	28–45	89.29–98.39%	HPLC-QqQ-MS	Szultka-Mlynska et al. (2018)
				85–135			
	NSAIDs (ibuprofen, diclofenac, naproxen, and nalidixic acid)	SPME- Fe_3_O_4_/Cu_3_(BTC)_2_ MOF	Human urine, serum, plasma, and tablets	0.03–0.05	94.0–102.0%.	HPLC	Mirzajani et al. (2019)
				0.12–0.18			
	Methamphetamine	SPME	Hair	0.067	90.2–95.8%	LC-MS	Meng et al. (2020)
	Amphetamine			0.067			
	Ketamine			0.067			
	Norketamine			0.067			
	Perphenazine, chlorpromazine, chlorprothixene, promethazine, and trifluoperazine	Hollow fiber SPME	Human whole blood and urine	0.025, 0.0125, 0.025, 0.025 and 0.0125	46.4–96.6% (blood)	UPLC-MS/MS	Li et al. (2020)
					65.2–101.9% (urine)		
	Alprazolam and amitriptyline	DI-SPME	Human blood and bone marrow	1.87–10.45	nr	LC-TOFMS	Majda et al. (2020)
	Bromazepam and carbamazepine, citalopram			5.60–31.35			
	Clonazepam,clorazepate, desipramine, and diazepam						
	Estazolam, flunitrazepam, and fluoxetine						
	Imipramine, lorazepam, lormetazepam, midazolam, nitrazepam, nordazepam, and nortriptyline						
	Paroxetine, prazepam, and temazepam						
	Tetrazepam, venlafaxine, and zolpidem						
	4-chloro-1-naphthol	SBSE	Human urine	0.034	87.4–141.3%	HPLC	Jillani et al. (2020)
				0.400			
	Tranexamic acid	SPME-Thin film	Human plasma and urine	10000–25000	nr	LC-MS/MS	Looby et al. (2021)
Others (food, animal tissue, *in vivo*, dosage forms)	Melatonin and other antioxidants	MEPS (C18)	Foodstuff	0.02	nr	HPLC-FLD	Mercolini et al. (2012)
	Carvedilol enantiomers	SBSE	Pharmaceutical dosage forms	8 (R) and 11 (S)	98–103%	HPLC	Taraji et al. (2015)
				25 (R) and 50 (S)			
	Clenbuterol	SPME	Pork	3.6 × 10^−6^	97.4–105.7%.	GC/MS	Ye et al. (2016)
	Salicylic, 3-methyl salicylic, 4-methyl salicylic, acetylsalicylic, and benzoic acids	SPME	Fruits and vegetables	2–28	78.0 ± 1.3%	HPLC	Aresta and Zambonin, (2016)
				7–95			
	Neurotransmitters	SPME	Macaque brain	25–20,000	80–100%	LC-MS/MS	Lendor et al. (2019)
	98 pharmaceutical analytes	SPME	Bovine tissue	(0.25–3X, where X corresponds to the MRL for each target analyte)	nr	SPME-DART-MS/MS	Khaled et al. (2020)

nr: not reported.

### Environment Applications

Many novel microextraction techniques are now replacing liquid extraction for the analysis of pharmaceuticals in water samples. It enriches and enables the direct injection of the analytes into the separation unit, which requires less solvents, time, and labor (Płotka-Wasylka et al., 2015; Ke˛dziora-Koch and Wasiak, 2018). They are usually coupled to gas chromatography–mass spectrometry or liquid chromatography–mass spectrometry. The determination of chlorophenols in water is very important due to its high toxicity level (Gallego et al., 2018). Many studies had reported the extraction of chlorophenols and some estrogenic compounds using HS-SPME, followed by on-fiber derivatization coupled with GC-MS (Yuan et al., 2017).

VOCs, being volatile, can be analyzed using preferably HS-SPME–based methods, rather than DI (DIN EN ISO 17943:2016). SPDE and ITEX-DHS are very useful for the VOC analysis in water, and due to the greater sorption volume, their linear range is broader and LODs are much lower. SPME-GC was developed for monitoring selected personal care products, estrogens, and pharmaceutical compounds (clofibric acid and carbamazepine) in surface water (Huang et al., 2015).

In a study to determine phenolic compounds, bisphenol A, and acidic pharmaceuticals, the sensitivity was adjusted by calculating the log D values of the target analytes at different pH levels. To ensure the desorption of strong polar–polar interactions between analyte and solid phase, SPME on a carbowax/templated fiber was used, and the results showed better sensitivity and remarkable improvement in the level of detection, 1 ng/ml in tap and river water and 2–9 ng/ml for wastewater (Kim et al., 2013).

Monolithic fibers have been developed and used to improve the mechanical stability of MIP to extract diacetylmorphine and analogous compounds in aqueous solution (Yılmaz et al., 2017). In another study, using multiple monolithic fiber SPME, analysis of sex hormones in tap and lake water was done with good performance and recoveries between 77.7 and 115%. The use of poly(1-allyl-3-methylimidazolium) bis(trifluoromethylsulfonyl)imide copolymerized with ethylene dimethacrylate as the monolithic fiber coat has resulted in a lifetime that is higher than 200 cycles and the analysis time is shorter (Liao et al., 2016).

MEPS provides a significant advantage by allowing the use of small sample volume, particularly when the amount of sample is limited, especially in forensic toxicology. The method showed a good recovery range for all the studied drugs of abuse 80–104%. In addition, the sorbent can be reused up to 80 times in human plasma (Fernández et al., 2017), the use of limited volumes, and the reusability of the sorbent can add to the green credit of the method (Mohamed, 2015). In another study, Soleimani and coworkers combined the MIP and MEPS (MIMEPS) and used it for the extraction of trans,trans-muconic acid; the LOQ of the method was lower than that suggested by the American Conference of Governmental Industrial Hygienists (75 μg/ml) (Soleimani et al., 2017). The MIMEPS used much smaller volumes of solvents for conditioning, washing, and elution procedures, which makes it a greener alternative than the conventional SPE technique (Soleimani et al., 2017). To enhance the extraction recoveries of sulphonamides from wastewater (88–109%), Salami et al. (Salami and Queiroz, 2014), used salting out by adding 15% NaCl, where the water molecules form hydration spheres around the NaCl molecules. In consequence, they reduce the water concentration available for dissolution of the analyte molecules, and this will drive additional analytes into the extraction phase.

### Biological Applications

The use of solventless microextraction techniques has a broad spectrum of applications in clinical control for diagnosis and treatment of diseases, doping, forensic analysis, and toxicology. All of the different techniques could be coupled to on-line HPLC, LC/MS, or GC/MS. Each technique as discussed earlier has its advantage and disadvantage. Table 2 represents some trials on extracting pharmaceuticals in the biological matrix with the extraction and analysis methods, recoveries, and LOD/LOQ.

Twenty illicit drugs and their metabolites were determined in oral fluid using MEPS; the method was successful in the determination of the selected drugs with fairly lower cutoff values than recommended by the Substance Abuse and Mental Health Services Administration (SAMHSA) (Montesano et al., 2015), using a miniaturized extraction procedure.

Analysis of amphetamine, methamphetamine, cannabinoids, cocaine, opioids, and hallucinogens was reported using various microextraction techniques. Determination was done in saliva samples for forensic purposes (Jain, 2017). The SPME coupled to mass spectroscopy and coated blade spray (CBS) was used to determine voriconazole in human plasma (Tascon et al., 2017) and immunosuppressant drugs in whole blood (Gomez-Ríos et al., 2018). Using the CBS has a challenge that the analytes largely bound to plasma proteins or red blood cells resulting in noticeably lower extraction recovery rates. Hence, it was treated by performing the analyte enrichment step through direct immersion in a solvent-modified matrix (Gomez-Ríos et al., 2018).

Abacavir, an anti-HIV drug, was determined using an SPME fiber coated with an acrylic acid–based MIP (Terzopoulou et al., 2016). An observed good recognition capability for abacavir in real sample analysis was reported, with extraction efficiencies 88–99%, and no matrix effects in either biological or environmental matrices—urine and wastewater.

Solid-phase microextraction (SPME) was also used for therapeutic drug monitoring of tranexamic acid in plasma and urine of patients with renal failure. The method was a greener alternative for the fast high-throughput monitoring of tranexamic with no biological matrix interference. The use of a biocompatible polyacrylonitrile-based hydrophilic–lipophilic–balance extraction phase help prevent the macromolecules from any interaction with the device which make the method convenient and easy to use as it allows direct exposure of the sample without major sample pretreatment steps which make it a greener alternative (Looby et al., 2021).

The use of FPSE for the determination of important biological molecules, 17-ethynylestradiol, β-estradiol, and bisphenol A, had shown lower detection limits of the analytes over previously reported methods. The analysis time was lower than the compared methods, and this contributes to the economic efficiency of the method and its greenness profile. Good recoveries were obtained for drinking water (96–98%), river water (92–94%), and groundwater (94–95%) (Kumar et al., 2014). In further study that determines some androgens and progestogens in water and urine samples, the analysis showed lower values for the matrix effect (between −10 and +7% for all the compounds), which ensures high selectivity from the interferences and the target analytes (Guedes-Alonso et al., 2016).

SPME coupled with thermal desorption–electrospray ionization/mass spectrometry (TD-ESI/MS) was used to study the pharmacokinetics of methylphenidate in plasma in a very small sample volume. High sensitivity and reproducibility of the SPME-TD-ESI/MS were reported due to the suitability of the PDMS for concentrating plasma methylphenidate, complete desorption in the thermal decomposition chamber, and the ionization efficiency of methylphenidate (Wang et al., 2018).

SBSE has been successfully applied to pharmaceutical analysis in different kinds of matrices, including environmental water, biological fluids, soils, and gaseous samples (Vo Duy et al., 2012; Hu et al., 2013). Several trials have been going on to improve the SBSE efficiency and its recent developments (Amlashi and Hadjmohammadi, 2016); nevertheless, the number of applications within this field is lower than that in food and environment analyses (Camino-Sánchez et al., 2014). This could be related to the complexity of the biological matrix where SBSE is not a highly selective or specific extraction technique. Moreover, many pharmaceutical analytes are polar compounds, and they have reduced extraction efficiency when PDMS is in the stationary phase (Camino-Sánchez et al., 2014; Taraji et al., 2015). Some approaches were carried out recently to increase the recovery of polar analytes, for instance, *in situ* derivatization and SBSE with solvent-swollen PDMS (Ochiai et al., 2018).

### Other Applications

Moreover, solventless extraction techniques can be used for pharmaceutical extraction in pharmaceutical formulations, animal tissues, and food samples, as shown in Table 2. Mercolini and coworkers (Mercolini et al., 2012) had determined melatonin and other antioxidants in food and beverages derived from grapes. The sample pretreatment was carried out by fast and reliable microextraction by packed sorbent (MEPS) procedure. This was followed by an HPLC determination coupled with fluorescence detection. Similarly, sodium chloride addition from 0 to 0.3 g/ml was used to enhance the extraction efficiencies of salicylic, 3-methyl salicylic, 4-methyl salicylic, acetylsalicylic, and benzoic acids from fruits and vegetables (Aresta and Zambonin, 2016).

SPME was used to determine clenbuterol in pork using GC-MS, with 97.4–105.7% recovery (Ye et al., 2016), and neurotransmitters in bovine tissue using LC-MS/MS (Lendor et al., 2019). SPME was used for direct analysis in real time, coupled to tandem mass spectroscopy, for rapid and high-throughput screening of multi-residue pharmaceuticals in bovine tissue. Although it has limited analyte scope and lower detectability levels compared to electrospray ionization–based methods, yet the high-throughput, simplicity, and real-time use are advantages of the technique. In another work (Khaled et al., 2020), the multi-residue pharmaceutical drugs in bovine tissue were analyzed using solid-phase microextraction and direct analysis in real-time-tandem mass spectrometry (SPME-DART-MS/MS).

SBSE was used to separate carvedilol enantiomers (Taraji et al., 2015) in pharmaceutical dosage forms. The applicability of two sorptive phases, poly(methyl methacrylate/ethyleneglycol dimethacrylate) (PA–EG) and polydimethylsiloxane, was evaluated for extracting carvedilol enantiomers from aqueous samples. The homemade PA–EG sorptive phase has shown recovery yields much better than the conventional PDMS and the least limit of detections and shorter time of analysis (Taraji et al., 2015).

## Conclusion

The extraction (cleanup) process is considered one of the major steps that hinder the greenness of any analysis procedure, especially complex matrices, due to high solvent consumption and use of toxic solvents; in addition, it prolongs the time of the analysis. Solid-phase extraction (SPE) techniques offer an interesting alternative to conventional liquid–liquid extraction. Having the advantage of being simple, economic in terms of time and solvents use, the SPE techniques have become more popular overtime. Advancement to improve them to be better solutions for sample preparation has been introduced, for instance, SPME, SBSE, MEPS, FPSE, and PAL SPME Arrow. These new techniques are considering the green analytical chemistry principles at many aspects (miniaturization, cost, solventless, etc.) that allow them to be a greener alternative.

Various factors contribute to select the optimum technique, for instance, the sample type, the analyte of interest, and the priority of the analysis. Moreover, the analytes’ characteristics, for instance, the partitioning constant, can affect the selection of headspace or direct immersion for the extraction mode.

Another advantage of these microextraction techniques is that they allow the analysis to take place using simpler analytical methods such as HPLC-UV/Vis while avoiding the use of more complex and expensive ones if needed, which again supports the GAC principles. Further investigation of more novel sorbents with advanced features that improve selectivity, loading capacity, or retention efficiency, and recoveries is still needed. In addition, exploring futuristic tools such as smartphones, microfluidics, and other small handheld detectors will be useful to ensure their sustainability and suitability.

## Abbreviations

CNTs, carbon nanotubes; DART-MS/MS, direct analysis in real-time tandem mass spectrometry; DI, direct immersion; DI-SPME, direct immersion–solid-phase microextraction; FPSE, fabric phase sorptive extraction; GAC, green analytical chemistry; GO, graphene oxide; HS, headspace; HS-SPME, headspace–solid-phase microextraction; IL-SPME, ionic liquid–solid-phase microextraction; ITEX-DHS, in-tube extraction–dynamic headspace; MEPS, microextraction in a packed sorbent; MMF-SPME, multiple monolithic fiber–solid-phase microextraction; MPI-SPME, molecularly imprinted polymer–solid-phase micro extraction; MWNTs, multi-walled nanotubes; PAL, prep and load solution; PILs, polymeric ionic liquids; SBSE, stir bar sorptive extraction; SPDE, solid-phase dynamic extraction; SPME, solid-phase microextraction; VOCs, volatile organic compounds.
